# Exploring students’ performance using lingua franca in science education: a study of grade ten students in Capiz, Philippines

**DOI:** 10.12688/f1000research.141170.1

**Published:** 2023-11-06

**Authors:** Dirk Diestro

**Affiliations:** 1 College of Education, Capiz State University, Roxas City, Western Visayas, Philippines

**Keywords:** Instruction, Language, Lingua Franca, Science, Students’ Performance

## Abstract

**Background:**

Currently global competitiveness is the main thrust of the country’s education department, raising the quality of education in the Philippines has become a priority for government officials, who see it as one way to address other teaching and learning challenges. Hence, this quasi-experimental research aimed to determine the influence of lingua franca-based and English-based instruction on the science performance of Grade ten students.

**Methods:**

The respondents of this study were forty-six Grade ten students out of the total population of fifty-three. Out of the forty-six respondents, twenty-three students were assigned to the experimental group and were taught using lingua franca-based instruction, and the remaining twenty-three were taught using English-based instruction. A researcher-made test was prepared and underwent validity and reliability tests. Mean was used to determine the science performance of the students, t-Test was used to test the significant difference between the students’ science performance before and after teaching using lingua franca-based instruction and English-based instruction, and Cohen’s d was used to compare two means in determining the effect size.

**Results:**

It was found that both groups exhibited fairly satisfactory science performance in the pre-test, and in the post-test, the control group reveals satisfactory Science performance while the experimental group shows very satisfactory science performance. No significant difference was determined between the pre-tests of both groups and significant differences were determined between the pre-test and post-test of both groups, and in the post-test of both groups, a large size effect was obtained in using the lingua franca-based medium of instruction on the science performance of the students.

**Conclusions:**

Hence, the practice of Lingua Franca-based instruction exhibits positive and desirable results in improving the performance of the students.

## Introduction

In many countries, the medium of instruction has long been a source of contention among educators, particularly in nations that were once subject to colonial rule. Although the nations gained their independence, their legacy still exists in one form or another. Language is the most notable of these legacies. The colonization made by the American regime in the Philippines left its traces in terms of language. In this present time where global competitiveness is the main thrust of the educative process, raising the quality of education in the Philippines has become a priority for government officials, who see it as one way to address other teaching-learning challenges.

The preference for English as the main medium of instruction in Philippine schools has left students from marginalized groups with little opportunity to learn and grow. Looking at the scenario nowadays, it is a known fact that students learn and acquire language differently, so it may come as easy as mimicking. While others need more time and tedious processes. With this, the challenge lies in the hands of the teacher. The teacher may either make or break a meaningful and fruitful teaching and learning process.

Language is a very important factor in the teaching-learning process since there is a mandate for the utilization of the medium of instruction. However, recent orders and memoranda present that students should be taught in a way that would suit the learners’ personal and national needs. There have been arguments and debates made in forums and seminars towards the best language of instruction in the teaching scenario and this gives a huge question in the field of the educative process that, could the utilization of bilingual education increase higher level learning and retention? Hence, this leads Philippine education to decide on the appropriate medium of instruction.

In the abovementioned scenario, the researcher contemplated the idea presented by Nisar and Ahmad (2011) that language is the medium of instruction by which all content in any discipline or level of learning is delivered to students. The question of which language should be used to teach particular subjects sparks controversy every time a new school system is put into place, since it challenges long-standing cultural norms Furthermore, the medium of instruction which refers to the language used holds a vital role in changing the teaching-learning process and making it easy or difficult for a student. The acquisition of learning among students is greatly influenced by their language since language is an important facet of culture. Therefore, it is considered an important instrument through which the change of cultural values is made easier and according to Amamio (2010), the individual develops his character from the perspective of his own cultural patterns, including languages.

In linguistics, the lingua franca refers to the alternative use of two or more languages in a single conversation. It is commonly used as a communication strategy among learners. Traditional methods or formal education often discourage lingua franca among students ( Setati, 2008). However, as Abad (2010) discussed, a lingua franca is a language that people use to communicate when they do not share one natively. All speakers of any language have learned how to code-switch or change their speech patterns according to the situation and environment in which they find themselves. In the academic world, lingua franca or code-switching is defined as the usage of a secondary language in addition to the primary language during speech. This can be seen most prominently in an educational setting—where students are encouraged and taught to switch between languages during class discussion.

From a general perspective, it is known that there are many factors that determine a student’s performance and language is one of those factors. Coherently, countries, where English serves as a second language, revealed that there is no single instructional strategy or methodology that can guarantee predictable success in class ( Chang et al., 2007). As a result, the focus of various research has shifted from instructional strategies and methodologies to language acquisition and utilization of English vis-à-vis native tongue in science classes ( Murcia, 2011).

The science performance of students at the secondary level according to the National Achievement Test shows a retrogressive pattern where the issues underpin the comprehension and analysis of the students ( Philippine Basic Education, 2019). With the aforementioned situation, the utilization of the lingua franca is presented by the Department of Education as mother tongue-based instruction to address the retrogressing science performance of the students and to cope with the demand for the UNESCO Education for All program.

In the presented idea of the Department of Education, it is believed that the official country’s language should be taught since it is a platform for learning information meaningfully to make students’ vocabulary wider in terms of understanding and comprehension. The utilization of lingua franca drives the students to have maximum performance since students actively participate during discussion and via their mother tongue, and students are able to actually learn the curricular content of instruction ( Thomas and Collier, 2007).

Going with the contemporary trend in education research and considering the above-mentioned scenario, the researcher was motivated to conduct a study that looks into the outcome of lingua franca-based instruction on students’ performance as compared to the typical practice of using English language-based science education.

This study determines the influence of lingua franca-based and English-based instruction on the science performance of Grade ten students. Specifically, it examines the level of science performance in each group at the start and end of the study, the significant difference between the pre-tests of the control and experimental groups, pretests and posttests of these groups, and between the posttests of these groups, and the effect size of lingua franca-based instruction and English-based instruction on the science performance of the students.

This study was anchored on the Experiential Theory of Kolb which stressed that the educational system should give opportunity to students to acquire and apply knowledge, skills, and attitude in a way that is relevant to their experiences ( Kolb, 1984; Satati, 2010). Applying the theory in this study, it can be assumed that students’ performance will be meaningful if the medium of instruction used is relevant to the language that they are using in their day-to-day lives.

## Literature review/study site

Lingua franca is a multilingual association of language used as a communication strategy among learners. In a multilingual society, language choice is heavily dependent on its sociolinguistic contexts including the location, participants involved, and the topic at hand ( Spolsky, 2004). Approximately, there are more or less seven thousand existing languages in the world which brings the idea of linguistic diversity ( Cenoz, 2013). As elaborated by Phillipson and Skutnabb-Kangas (2017), linguistic diversity is needed as it reflects the individual’s community culture, tradition, and identity.

The Philippines’ K to 12 Basic Education Curriculum includes a mandate for mother tongue-based instruction, beginning in kindergarten and continuing through third grade. This supports the goal of having all children, regardless of background, become readers and writers by the end of third grade. Although there is an argument about the use of English as a medium of instruction versus mother tongue-based instruction in consonance with the multilingual education, the trend among teachers nowadays is teaching their students using mother tongue-based instruction. Students who were taught using mother tongue-based instruction tend to think critically and were able express themselves with greater fluency. One of the main reasons for this policy is that some people believe children learn faster when they are taught by teachers who use a form of language with which they are familiar at first. Because native and regional languages help improve children’s language skills and strengthen their social awareness, teaching materials for bilingual learners should be in such languages ( Valerio, 2013).

Quijano (2010) underscores that reading fluency and comprehension were lowest among those students taught to read only in English or Filipino. Hence, multidisciplinary studies have been carried out to determine the best approach to teaching students a new language: either through their native tongue or by first learning an established international language such as English before using their own. Several case studies were conducted in the Philippines over the years on mother tongue-based multilingual education and these include the Iloilo Experiments, the Rizal Experiment, and case studies of other components such as Lingua Franca Project in Ifugao Province. The aforesaid studies have shown that when teachers use students’ native language as the instructional medium, those students who read with inclination have a greater chance of developing the skills necessary to succeed in science, math and other related fields. They also tend to do better in classes where their native language is used.

As revealed by Nolasco (2009), children with a strong background in their native language can more easily pick up other languages and when children begin school with their first language as the foundation for further learning, with an understanding of the connections between these two languages—which allows for a smooth transition into learning new subject matter—they tend to emerge as more competent learners overall. Reports on the Philippine educational system tend to emphasize weak English, science, and mathematics skills among Filipino students. Mother tongue-based multilingual education seeks to address the high functional illiteracy rate in the Philippines and its consequences—the high dropout and noncompletion rates among students.

Evidently, during high school education, classes are taught in Filipino and English, but teachers use their mother tongue as a way to explain concepts when necessary ( Licuanan, 2007). This paves the way for the crafting and implementation of DepEd Order No. 74, series of 2009—Institutionalizing Mother Tongue-Based Multilingual Education (MLE) which replaces the Bilingual Education Policy with its assertion that research has shown that students in local communities and around the world are more likely to succeed when they learn using their mother tongue as a primary language of instruction. Further, Nolasco (2009) stresses the suitability of multilingual education in the learning process by writing an article in Education’s Primer underpinning that MLE starts from where learners are, and from what they already know: “The strategy is to develop cognitive skills of learners in their first language—and then transfer them into the medium of instruction.” It has been demonstrated in numerous surveys, proficiency tests, and in the complaints of businesses that corporate employers have not found the Bilingual Education Policy as effective as originally hoped. According to Quijano (2010), a language’s efficiency is directly related to how often it is used, for example, Cebuanos prefer to use English instead of Filipino, which negatively affects proficiency in the latter. Although today’s children are generally fluent in oral Filipino, their fluency in writing and reading the language is limited. This problem becomes even more acute when it comes to English—the country’s official national language.

On the other hand, Quijano (2010) disputes multilingual education implementation by noting that childrens’ poor command of the English language is a result of their limited exposure to it aside from the books, magazines, and other instructional materials available in schools and libraries that are written in English. As a result, language-competence problems such as poor reading comprehension have arisen among students. Licuanan (2007) points out that to combat the dwindling proficiency of Filipino English speakers, efforts have been made to improve their command of the language. However, while the push for English as a primary medium of instruction may seem like an effective strategy to solve this problem, in fact language experts have noted that it might simply be an extension of the myth that if something is good then more will be even better. The deterioration of English must be understood in the context of the overall decline in Philippine education. This is not a problem limited to English— students are also performing poorly in math and science classes.

Walter (2008) noted that many of the arguments against taking language into consideration in educational policies for developing nations have little to do with effectiveness. Thus, he pointed out in his findings that people often have a number of reasons for not learning another language. One reason is that they think it will give other people an advantage over them. Another is that they think it will be easier to build a country if everyone speaks the same language. Some people also do not want their children to learn languages other than one they already know, because they think it will confuse their children. Some people believe that if you speak a certain language, you should be able to use it in school and at work, which means that it has to be developed before it can be used as a medium of instruction. Some people think that if there are not many people who speak a certain language in one place, it won’t be worth teaching it there. Some people also believe that if there are not any jobs using a particular language available right now, there would not be anytime soon either.

In bilingual classrooms, as highlighted by Baker (2009), students often switch between different languages. Teachers regularly use two languages in a bilingual classroom without official backing from policymakers. They integrate these languages to achieve their teaching tasks. On the contrary, Jacobson (2009) argues that integrating both languages in the classroom is beneficial. He says that teachers should not randomly switch from one language to another, translate, preview, or review material, or use purposeful concurrent language use. However, Baker (2009) claimed that there are many language-switching cues that cause a speaker to shift languages. These include reinforcement of concepts, reviewing material, capturing students’ attention, and changing topics. Other cues are gaining rapport with students and changing from formality to informality. Translanguaging can create opportunities for deeper learning, language enrichment and collaboration between home and school.

## Methods

### Purpose of the study and research design

This study employed a quasi-experimental research design in determining the influence of lingua franca-based and English-based instruction on the science performance of Grade ten students using the pretest-posttest non-equivalent control design. In the pretest-posttest control group design, the experimental group received treatment that was withheld from a control group. Before the introduction of the intervention, surveys and tests were conducted in both experimental and control groups last October 24, 2022. After the intervention was introduced to the experimental group, a post-test and observation of both groups took place last January 13, 2023.

### Participants

The respondents of this study were identified and grouped by employing matched pairs design to address the issue of “collective similarity,” and this was employed by matching the respondents in terms of their first grading grade in Science. The participants of this study were forty-six (46) Grade ten students from Capiz State University – Roxas City Main Campus, Laboratory High School out of the total Grade ten population of fifty-three (53) students. Out of the forty-six (46), 23 students were assigned to the experimental group and were taught using lingua franca as the medium of instruction, and the remaining 23 were taught using English as the medium of instruction. The respondents together with their parents or guardian were invited for the orientation of the purpose, preliminary plans and commencement of the study last May 24, 2022. The researcher sought their approval through a parental permission form.

### Data collection instruments

The data gathering instrument of this study was initially made with 100-item researcher-made tests encompassing the topics in the third quarter specified in the curriculum guide for Grade ten science. The test questions were subjected to a reliability and validity process. After the validity process, pilot testing was conducted utilizing the Grade ten students from other schools who were not included as participants in the experimentation. After the pilot testing, the researcher subjected the 100-item researcher-made test to item analysis, difficulty, and discrimination index analysis to establish whether the items will be retained, revised, or rejected. As a result, from 100 items, only 60-item were accepted. The 60 items used in the pre-test and post-test were rearranged in the post-test. To interpret the scores obtained by the participants, the researcher used the arbitrary scale of Outstanding (48.51 – 60.00), Very Satisfactory (36.51 – 48.50), Satisfactory (24.51 – 36.50), Fairly Satisfactory (12.51 – 24.50), and Did Not Meet Expectations (0.00 – 12.50).

### Data collection procedures

*Pre-experiment phase*

The researcher prepared the list of topics, daily lesson plan, and researcher-made test for the pre-test and post-test before conducting the research study. Also, during this phase, pilot testing was conducted. In addition, phase validity of lesson plans and the researcher-made test were done. Further, the researcher oriented the participants about the purpose of the study. After those significant steps, the researcher was ready in employing the intervention. A copy of the pre-test and post-test can be found under *Extended data* ( Diestro, 2023).

*Experiment phase*

As the experimentation commenced, the experimental group was taught using lingua franca-based learning as the medium of instruction, and the control group was taught using English-based learning as the medium of instruction. At the start of each lesson, the researcher administered a pre-test and proceeded to the delivery of the lesson wherein the entire teaching was conducted using the lecture-discussion strategy and differed only on the medium of instruction used by the researcher in the respective groups. After the delivery of the lesson, the researcher administered a post-test utilizing the researcher-made test used in the pretest however; the test item placement in the pre-test was rearranged in the post-test.

*Post-experiment phase.*

At the end of the experimentation, students in the experimental group who were taught using lingua franca-based instruction were asked about their comments or reactions with regard to the medium of instruction employed. Additionally, during this phase, the scores of the students for both groups were recorded, tabulated, and analyzed using the appropriate statistical tools.

### Statistical data analysis

Mean was used in determining the science performance of the students taught using lingua franca-based instruction and English-based instruction, t-Test for paired samples was utilized to determine the significant difference between the students’ science performance before and after teaching using lingua franca-based instruction and English-based instruction and this inferential test was set at 0.05 alpha level of significance, and Cohen’s d was used when comparing two means to determine the effect size.

## Results and discussion

### Science performance between groups in the pre-tests and post-tests

The results reveal that prior to the introduction of the intervention, the control group revealed “fairly satisfactory” (m = 17.04) science performance similar to the experimental group (m = 18.96). Moreso, after the intervention, the scores obtained in the post-test of the control group revealed “satisfactory” (m = 31.57) science performance and the experimental group showed “very satisfactory” (m = 42.17) science performance. This implies that the prior knowledge of the control and experimental groups towards that subject matter and competencies to be taught was the same. Also, this indicates that there was homogeneity of the scores obtained by students in the pre-test. The full raw data can be found under *Underlying data* ( Diestro, 2023).

On the other hand, the results of the post-test revealed that regardless of whether lingua franca or English were used as the medium of instruction, students improved their science performance. However, the increase in performance was in favor of the lingua franca-based medium of instruction which exhibits a favorable effect in improving the science performance of the students. Moreso, the favorable improvement of students in the experimental group may be due to the openness of collaboration and exchange of ideas among learners and between teacher and students using lingua franca as the medium of instruction where understating and retention were very evident since students turn active and do respond more to the topics taught. The results of this study conforms to the study of Bernardo (2005) whose findings show that multi-lingual education could be used in facilitating students’ learning and achievement since the learning process is flexible and best appropriate for code-switching. In addition, Amamio (2010) reveals that people acquire their personalities through their interactions with the world around them, including other people and language which affirms the result of the study.

### Difference in the science performance between groups in the pre-test

The result discloses that there was no significant difference between the pre-tests of both the control and experimental groups (p-value = .053) as shown in Table 2. This implies that prior to the start of the conductance of the intervention, students assigned to both control and experimental groups possess the same level of science performance which showed that both groups were very suitable for comparing the effect of lingua franca and English as mediums of instruction. The result of this study corroborates with the study made by Marzban et al. (2013) whose result reveals that the homogeneity of the participants was obtained before introducing experimentation between experimental and control groups to attain reliable results in determining the effect of different strategies on the reading comprehension of the learners.

**Table 1.** Science performance between groups in pre-tests and post-tests.

Group	Test	Mean	Description
Control	Pretest	17.04	Fairly satisfactory
	Posttest	31.57	Satisfactory
Experimental	Pretest	18.96	Fairly satisfactory
	Posttest	42.17	Very satisfactory

**Table 2.** Difference in the science performance between groups in the pre-test.

Test	Group	Mean	SD	T-value	df	Sig
Pretest	Control	17.04	3.27	(-)1.99	44	.053
	Experimental	18.96	3.27			

### Differences in the science performance between groups in the pre-tests and post-tests

The result shows that there were significant differences between the pre-test and post-test of the control group (p-value = .000), and of the experimental group (p-value = .000) as reflected in Table 3. The results imply that using lingua franca or English as the medium of instruction improves the science performance of the students. Further, the results infer that students in the control group improved their performance as reflected in Table 1 where the performance in the pre-test was “fairly satisfactory” which turned to “satisfactory” in the post-test. This means that students acquired new learning during the time of experimentation using English-based medium of instruction since the teaching-learning process took place where student participation, the art of questioning, effective teaching strategy, and assessment were all adhered. The result of this study affirms Delos Santos’ (2013) findings which discloses that the pre-test and post-test of the control group exhibited significant difference which indicates that students improve their mathematical performance from moderately high to high.

**Table 3.** Differences in the science performance between groups in the pre-tests and post-tests.

Group	Test	Mean	SD	T-value	Df	Sig
Control	Pretest	17.04	3.27	(-)14.01	22	.000
	Posttest	31.57	4.46			
Experimental	Pretest	18.96	3.27	(-)26.18	22	.000
	Posttest	42.17	2.52			

On the other hand, students in the experimental group also exhibited improvement in their science performance as shown in Table 1 which entails that the pre-test resulted in “fairly satisfactory” and after the intervention using lingua Franca-based instruction resulted in “very satisfactory” and it shows greater effect in terms of science performance. The said increase can be attributed to the reason that students felt comfortable explaining and expressing their idea using a combination of language where the students may abruptly shift to give emphasis on their point. This study conforms to the study of Gardner (2001) that the use of lingua franca helps students understand what is taught and lessen the difficulties of understanding the lesson which leads to the improvement of the performance of the students. More so, Warden *et al.* (2004) reveal that the medium of instruction affects learners’ scholastic performance.

### Difference in the science performance between groups in the post-tests

The results show that there was a significant difference between both groups in the post-test (p-value of.000) in favor of the experimental group as reflected in Table 4. The result infers that the students assigned to the experimental group obtained high performance in science compared to those students assigned to the control group and this can be attributed to the belongingness that the students felt during the discussion since developing one’s sense of belongingness inside the classroom makes learning meaningful and easy for the students to acquire and retrieve knowledge since the reinforcement to the learning that took place was experienced-based. Moreso, using a combination of language helps the students facilitate comprehension and deep analysis during class discussions. The result of this study affirms the findings of Libutaque (2001) which reveals that the use of primary and secondary languages of the students in teaching math and science facilitates learning well. Furthermore, the results disclosed by Clarckson (2002) show that students who were fluent in both their first and second language demonstrate outstanding cognitive processes which result in successful learning which also conforms with the results of this study.

**Table 4.** Difference in the science performance between groups in the post-tests.

Test	Group	Mean	SD	T-value	df	Sig
Posttest	Control	31.57	4.46	(-)9.34*	44	.000
	Experimental	42.17	2.52			

### Effect size of lingua franca-based instruction

The obtained Cohen’s d value of 2.93 as shown in Table 5 infers that there was a large size effect obtained on the science performance of the students using lingua franca as the medium of instruction. This implies that the lingua franca-based medium of instruction when used in science classes, may surely exhibit positive and desirable results in improving the performance of the students given that using lingua franca as the medium of instruction motivates students’ to actively respond and inquire, ensures rapport towards students, promotes better understanding towards word meaning and maintains active teaching-learning process atmosphere in the classroom. The results of the study corroborates the findings of Delos Santos (2013) which showed lingua franca promotes students’ understanding due to familiarity with the medium of instruction used during the discussion. Also, according to Probyn (2012), it was identified that some teachers use the home language as a tool for scaffolding since these teachers believe that first language utilization is the way to accelerate second language acquisition which corroborates the results of this study.

**Table 5.** Effect size of lingua franca-based instruction.

Test	Group	Mean	SD	Cohen's d
Posttest	Control	31.57	4.46	2.93
	Experimental	42.17	2.52	

## Conclusion

It was found that in the pre-test, both groups displayed “fairly satisfactory” science performance. Also, the scores obtained in the post-test of the control group reveals “satisfactory” science performance and the experimental group shows “very satisfactory” science performance after subjecting the participants to the intervention. Hence, the respondents in both control and experimental groups have only a satisfactory knowledge of the topics to be taught during the conduct of the intervention. After the intervention, the post-test scores reveal that there was an improvement in science performance among participants in both control and experimental groups. Thus, whether lingua franca or English as the medium of instruction is used in the classroom, students may improve their science performance. However, the increase in performance was in favor of the lingua franca medium of instruction. Lingua franca-based instruction in science facilitates understanding and stimulates active participation in the class discussion. Therefore, employing lingua franca as the medium of instruction exhibits a favorable effect in improving the science performance of the students.

There was no significant difference between groups in the pretests. Therefore, students were all in the same level of science performance which reveals that the participants have the same level of knowledge acquisition before the study.

There were significant differences between groups in the pretest and posttest. Thus, this concludes that the use of lingua franca exhibits favorable dominance compared to English-based instruction in improving the science performance of the students. Moreso, students’ interests increase since the utilization of language allows them to express their ideas freely and understand the topic well.

There was a significant difference between groups in the post-test in favor of the experimental group. Therefore, the students assigned to the experimental group were able to increase their understanding, acquisition of knowledge, and learning retention due to the language used.

There was a large size effect obtained in using the lingua franca-based medium of instruction on the science performance of the students. Therefore, the usage of lingua franca-based instruction exhibits positive and desirable results wherein students taught using lingua franca improve their performance significantly better compared to the other group.

## Ethical approval

The researcher secured permissions from the University President, Campus Administrator, Dean, and Program Chairperson to carry out the study. The ethical approval letter for this study was acquired from the University President and Campus Administrator of Capiz State University dated May 20, 2022. Following the approval, participants were informed about the study’s objectives and provided their consent for the utilization of data.

## Data Availability

Figshare: Exploring Students’ Performance Using Lingua Franca in Learning Science. https://doi.org/10.6084/m9.figshare.22801796.v1 ( Diestro, 2023). This project contains the following underlying data:
• Raw Data Lingua Franca.xlsx ( *file contains the scores of the students treated as science performance of the participants*) • Statistical Analysis Tables Lingua Franca.xlsx ( *file are the tables for interpretation presented in the results and discussion in the manuscript*) • Statistical Analysis Lingua Franca.doc ( *file contains the statistically treated data from the SPSS*) This project contains the following extended data:
• POST TEST Exploring Students’ Performance Using Lingua Franca in Learning Science.pdf • PRETEST Exploring Students’ Performance Using Lingua Franca in Learning Science.pdf Data are available under the terms of the Creative Commons Attribution 4.0 International license (CC-BY 4.0).
