# Supplementary material for: Exploring students’ performance using lingua franca in science education: a study of grade ten students in Capiz, Philippines
Source: F1000Res. 2023 Nov 6;12:1439. [Version 1] doi: 10.12688/f1000research.141170.1 (PMC10988198; doi:10.12688/f1000research.141170.1)
Supplement: Peer review history

# Reviewer report: Reviewer response for version 1

**DOI:** 10.5256/f1000research.154587.r250138

**Published:** 2024-04-02

**Authors:** Salah Ben Hammou

**Affiliations:** 1 University Hassan 1st, Settat, Chaouia-Ouardigha, Morocco

Drawing on quasi-experimental research design, the study investigated 10th grade students' science performance level in two different MOI (medium of instruction environment) environments: Lingua franca-medium instruction and English-medium instruction. The findings showed that both groups (the experimental and control groups) achieved satisfactory results in the post tests.  

 Revisions required: 

**Abstract: **

1. Justify the use of quantitative research design for this study in one sentence.
2.  Provide a sentence on the pedagogical implication of the study.

 
**Introduction:**

1. The reader would want to know the objective(s) of the study at this stage.
2. A paragraph on the significance of the study and the gap the author is trying to bridge in the existing literature is also advisable at this stage.

***Review of literature***

 1. This section needs consideration. A s a reader, I expected the author to report similar previous findings on the impact of Lingua franca-based instruction and EMI on students' performance in science from different context worldwide. 

 2. including updated existing research on the topic is advisable. 

***Methodology: *** Explain the choice of the quasi-experimental research design? why not mixed methods design?

**Results**

 1. I suggest separating the section results and discussion. 

 2. Did the author follow any procedures to ensure the accuracy and reliability of the datasets.

 3. Perhaps the author should relate the results to the research questions.

**Discussion**

 Avoiding repeating the results presented in the previous section. The reader would want a stronger discussion which answers to the research questions by contrasting the previous studies (especially those from the local context) with the new data gathered.

Is the work clearly and accurately presented and does it cite the current literature?

Partly

If applicable, is the statistical analysis and its interpretation appropriate?

Yes

Are all the source data underlying the results available to ensure full reproducibility?

No

Is the study design appropriate and is the work technically sound?

Yes

Are the conclusions drawn adequately supported by the results?

Partly

Are sufficient details of methods and analysis provided to allow replication by others?

Yes

Reviewer Expertise:

Applied Linguistics, EMI, CLIL, Bilingual education

I confirm that I have read this submission and believe that I have an appropriate level of expertise to confirm that it is of an acceptable scientific standard, however I have significant reservations, as outlined above.

# Reviewer report: Reviewer response for version 1

**DOI:** 10.5256/f1000research.154587.r250151

**Published:** 2024-03-11

**Authors:** Balachandran Vadivel

**Affiliations:** 1 Cihan University-Duhok, Kurdistan Region, Iraq

While the study provides valuable insights into the potential benefits of lingua franca-based instruction, there are a couple of potential limitations worth considering. Firstly, the sample size of only forty-six Grade ten students from a single school may limit the generalizability of the findings. A larger and more diverse sample across multiple schools could provide a more comprehensive understanding of the effects of instructional language on science performance. Additionally, the study could have benefited from a longer-term follow-up to assess the sustainability of the observed improvements. Without longitudinal data, it's difficult to determine whether the enhanced performance seen immediately after the intervention persists over time or if it diminishes. These limitations should be addressed in future research to ensure the validity and applicability of the findings beyond the specific context of this study.

Is the work clearly and accurately presented and does it cite the current literature?

Yes

If applicable, is the statistical analysis and its interpretation appropriate?

Yes

Are all the source data underlying the results available to ensure full reproducibility?

Yes

Is the study design appropriate and is the work technically sound?

Yes

Are the conclusions drawn adequately supported by the results?

Yes

Are sufficient details of methods and analysis provided to allow replication by others?

Yes

Reviewer Expertise:

English Language Teaching, Second Language Acquisition, Applied Linguistics and Educational Psychology.

I confirm that I have read this submission and believe that I have an appropriate level of expertise to confirm that it is of an acceptable scientific standard.

# Reviewer report: Reviewer response for version 1

**DOI:** 10.5256/f1000research.154587.r229977

**Published:** 2024-01-25

**Authors:** Sohaib Alam

**Affiliations:** 1 Prince Sattam bin Abdulaziz University, Al Kharj, Riyadh Province, Saudi Arabia

Dear Author,

 This paper has focused on an interesting and innovative topic. However, some major issues need clarification and additional work. I have to say, if some serious problems are not properly addressed.

 1. What are the findings?

 2.  How did these

 motivate the researchers to conduct this study?

 3. Where are the findings? There should be concrete findings of your study.

 4. The major concern is no baseline provided for further data analysis. Were there statistically significant differences in prior achievement between the two groups? If the experiment group performed better than the control group at the beginning, it is not robust to imply that an intervention was effective in promoting student achievement. The authors would like to check their data and answer this with caution.

 5. Please cite related literature of last five years.

Is the work clearly and accurately presented and does it cite the current literature?

Partly

If applicable, is the statistical analysis and its interpretation appropriate?

Partly

Are all the source data underlying the results available to ensure full reproducibility?

No

Is the study design appropriate and is the work technically sound?

Partly

Are the conclusions drawn adequately supported by the results?

Yes

Are sufficient details of methods and analysis provided to allow replication by others?

Yes

Reviewer Expertise:

Pedagogy and Education ,Teaching and Learning, Pedagogy, Learning, Collaborative Learning, Blended Learning, Cooperative Learning, ICT in Education

I confirm that I have read this submission and believe that I have an appropriate level of expertise to confirm that it is of an acceptable scientific standard, however I have significant reservations, as outlined above.
